# The Genetics of Racing Performance in Arabian Horses

**DOI:** 10.1155/2019/9013239

**Published:** 2019-09-02

**Authors:** K. Ropka-Molik, M. Stefaniuk-Szmukier, A. D. Musiał, B. D. Velie

**Affiliations:** ^1^Department of Animal Molecular Biology, National Research Institute of Animal Production, Poland; ^2^Department of Horse Breeding, Institute of Animal Science, The University of Agriculture, Krakow, Poland; ^3^Faculty of Biochemistry, Biophysics and Biotechnology, Jagiellonian University, Poland; ^4^University of Sydney, School of Life and Environmental Sciences, Sydney, Australia

## Abstract

Arabian horses are commonly believed to be one of the oldest and most influential horse breeds in the world. The high financial benefits obtained from races tend to search for genetic markers strongly correlated with the results achieved. To date, the modern approaches such as transcriptome, miRNAome, and metabolome analyses have been used to investigate the genetic background of racing performance as well as endurance capacity in Arabians. The analysis of polymorphisms at the genome level has also been applied to the detection of genetic variants associated with exercise phenotype in the Arabian breed. The presented review summarizes these findings, with a focus on the genetics underlying flat racing and endurance performance traits in different Arabian horse populations.

## 1. The Arabian Horse

**Figure 1:**
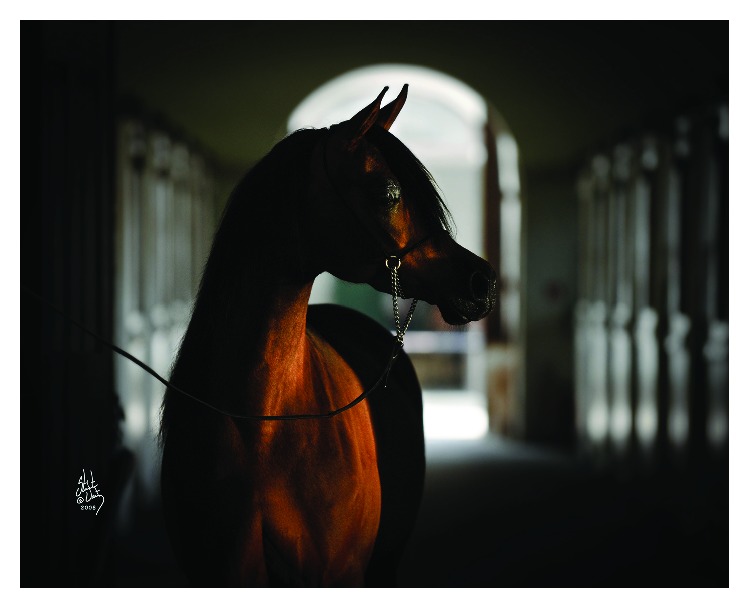
The Arabian mare—Pianissima (Gazal al Shaqab-Pianosa by Eukaliptus) from Janów Podlaski State Stud in Poland (double triple crowned) (photo credit Stuart Vesty; all rights reserved).

**Figure 2:**
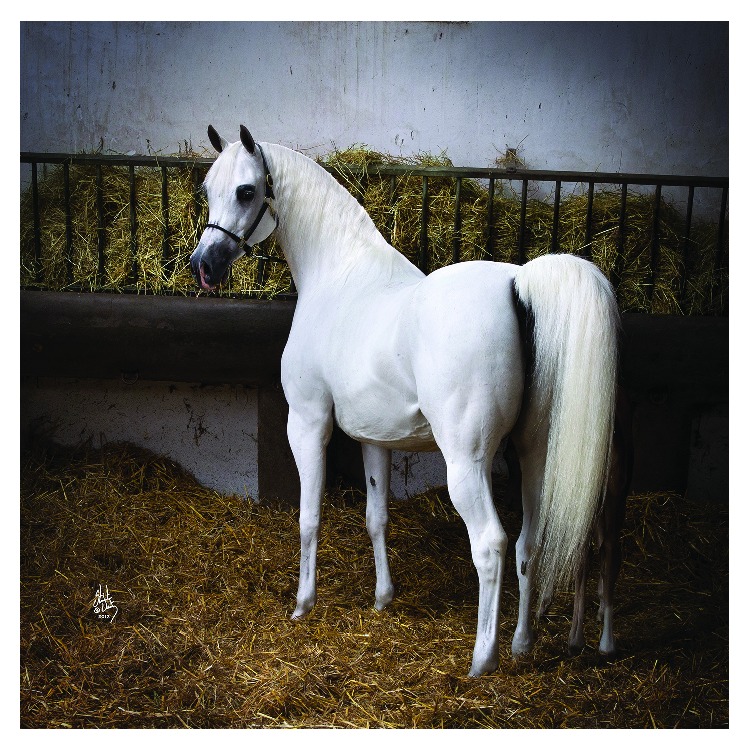
The classic grey Arabian mare at the barn (photo credit Stuart Vesty; all rights reserved).

Arabian horses are commonly believed to be one of the oldest and most influential horse breeds in the world. Widely recognizable due to their unique beauty and appearance, Arabian horses also possess many structural and functional adaptations for athletic performance with Arabian horses known worldwide for their extraordinary stamina (Figures 1 and 2). While Arabian horses often compete in a wide variety of equestrian sports, they tend to dominate endurance competitions. As a result, Arabian horses are often used to enrich not only refinement in other horse breeds but also endurance abilities [1].

At present, the best-known Arabian horse populations are from the Middle East (including Straight Egyptian), Europe (Polish Arabians and French Arabians), the United States, and Canada. However, studies performed using mitochondrial DNA (mtDNA) confirmed that Arabians originate from the Middle East with current populations in the Middle East, Europe, and North America demonstrating a high number of similar haplogroups and relatively low maternal genetic diversity [2–4]. Despite this close relationship, selection criteria for horses in these regions can often differ. Polish Arabians are generally characterized by unusual beauty as well as desirable racing performance features with race track performance being one of the main criteria for selection. Breeding evaluations for Polish Arabians are typically based on type (i.e., appearance), proper conformation, and racing prowess [5, 6]. On the other hand, the French population is recognized mainly as a race horse, with Middle Eastern lines representing selection that typically favours appearance traits.

Nevertheless, selection in Arabian horses has historically focused on maintaining the breed's usability together with preserving a desirable type. As a result, although widely viewed as endurance-activity horses, it is not uncommon for 2.5- to 5-year-old horses to be introduced to flat race training and racing prior to undergoing endurance training [7]. Once an Arabian horse's flat racing career is completed, most horses are then used for breeding selection, with some horses subsequently introduced to endurance training. Selected horses, typically 5 to 7 years of age, are prepared for moderate distances of up to 80 kilometres. Horses demonstrating sufficient predisposition and adequate age (7 plus years) are then regularly trained for participation in long-distance events (120-160 km).

## 2. Differentiating Arabian Horse Performance from the Performance of Other Racing Breeds

Compared with other race horse breeds, the muscle tissue of Arabian horses is characterized by significant differences in structure—the predominance of oxidative fibre type I is observed in Arabians, while a higher proportion of fibre type IIa is detected for example in Thoroughbred (TB) horses [8, 9]. This variation in fibre type distribution results in different performance traits. In an Arabian horse's muscle, the higher proportion of oxidative type I fibres (characterized by a low glycogen content and high triglyceride storage capability) results in a greater use of fat for energy [10]. In endurance athletes, an increased aerobic capacity allows skeletal muscle to metabolize more fat and use energy from fatty acids, while at the same time using carbohydrates as an energy source [11]. The aerobic conditioning programme in endurance-type athletes induces an increase in the activity of oxidative metabolism and a decrease in anaerobic metabolism. Similarly, in Arabian horses, during intensive training, the cellular metabolism in muscle tissue is switched from aerobic glycolysis to lipid metabolism due to the different fibre type distributions that determine distinct metabolic responses. Prince et al. [10] reported higher free fatty acid concentrations and utilization in Arabian horse blood compared to that of Thoroughbred horse blood during exercise. While during exercise in both breeds, a shift towards oxidizing more fat and less glucose occurs, yet in Arabian horses these changes are more evident and significantly greater. Such results indicated significant differences in effort and various racing predispositions observed in racing breeds, which are most likely conditioned by different genetic backgrounds. The adaptive response to exercise is associated with changes in gene expression, metabolism, muscle cell cycle progression, and protein homeostasis. However, the exact mechanisms that occur in equine muscles during exercise related to skeletal muscle endurance in high-intensity training are not well understood.

## 3. Heritability of Racing Performance in Arabian Horses

In Arabian horses, the heritability for racing time in flat races has been estimated between 0.175 and 0.304, with moderate to high repeatability (0.295-0.460) [12]. Moreover, Orhan and Kaygisiz [13], analysing Turk-Arabian horses, estimated that the heritability of race finishing times ranged from 0.11 to 0.44. The authors concluded that for racing time, moderate genetic progress would be possible if selection considered the phenotypic value of the horses, especially as it pertains to short- and medium-distance abilities. The possibility of achieving the best results in short or long-distance races may indicate different genetic conditioning on the basis of the optimal race distance predisposition. A research performed on a Polish population of Arabian horses also showed strong genetic correlations for placings recorded depending on race distances (from 0.95 to 0.99) [14]. However, all of the aforementioned studies focused on the heritability of flat racing traits, which tends to explain sprint performance but does not provide any specific information about endurance performance.

Consequently, Younes et al. [15], examining results of more than 100 endurance races, showed that the highest heritability for cardiac recovery time was 0.46, while speed and heart rate recovery were, to a large extent, conditioned by environmental circumstances. In a French population of Arabian endurance horses, Ricard and Touvais [16] estimated a moderate heritability for speed at 0.28 with repeatability at 0.44 and a low heritability for placing (0.06). The authors also indicated that for shorter distances (20-60 km), conditioned younger horses were similar to adult horses (to 160 km), which can create a problem in obtaining a reliable genetic correlation value for an established precocious criterion. The heritability values for flat racing and endurance performance clearly indicate the possibility for genetic progress of both types of Arabian horse performance. However, selection would likely need to be focused on improving each type of exercise phenotype independently.

## 4. Genome Scan Analysis

In 2017, Ricard et al. performed a genome-wide association analysis (GWA) to identify mutations related to endurance racing performance in a French population of Arabians and Arabian-cross horses. According to a global association analysis of SNPs and racing results (total race distance, average race speed, and finishing status), the authors selected five quantitative trait loci (QTL) related to endurance racing performance. Two genes were selected, SORCS3 on chromosome 1 and SLC39A12 on chromosome 29, as the strongest and the most significantly correlated with the analysed traits. The genome-wide screen for endurance exercise ability enabled the detection of a new candidate equine long noncoding RNA (KCNQ1OT1) potentially related to cardiac rhythm regulation. Moreover, the authors found three intergenic SNPs, which were proposed to be markers of key genomic regulatory functions in response to stress from endurance exercise. The study provided a strong basis for further analyses investigating the genetic background for stamina and endurance characteristics in Arabian horses.

Gurgul et al. [17] identified selection signatures in Arabian horses most likely associated with selection focused on improving endurance riding and racing. Based on SNP information obtained using Neogen Equine Community BeadChip assay (Illumina), authors detected several selection signals on horse chromosome (E. caballus, ECA) 1, 3, 11, 15, 17, and 22. Genes identified were associated with adaptation to effort such as ATP synthesis coupled electron transport (COX4I1), vascular smooth muscle contraction (ADCY1), taurine and hypotaurine metabolism (GAD1), oxidative phosphorylation (COX4I1), and insulin signalling (CBLB) as well as secretion (ADCY1) pathways. Of particular note from this recent investigation is the genomic region within the SLC16A1 (Solute Carrier Family 16 Member 1) gene. A previous study had already demonstrated a significant association of SLC16A1 gene with adaptation to exercise via controlling the lactate metabolism during flat racing competition in Arabian horses with the more recent study by Gurgul et al. providing further support that the region is likely under selection pressure in Arabian horses [17, 18].

## 5. Transcriptomic and miRNAomic Analyses of Racing Performance in Arabian Horses

The exact response to long-term training in preparation for flat racing has also been widely investigated in a Polish population of pure-breed Arabian horses [18, 19]. Previous studies were aimed at identifying all of the molecular adaptation mechanisms occurring in blood and skeletal muscle that could predispose Arabian horses to flat racing (effort type is more “sprint” than “endurance”) success. Although selection in Arabian horses is focused on improving stamina and athleticism to obtain the best results in endurance racing, Arabian horses often first compete for at least one flat racing season before fully maturing and moving on to endurance training. However, when horses achieve outstanding results in flat racing, these animals tend to compete for more than one season and will not compete in endurance races. As horses predisposed to endurance effort often cannot obtain success in flat racing, different molecular backgrounds are expected to determine exercise phenotype.

Researches performed in pure-breed Arabian horses during training for flat racing have allowed for the detection of the most overrepresented genes and pathways essential for adaptation to flat racing [18, 19]. The results indicated that in Arabian horse muscle, long-term exercise induced the expression of genes related to fatty acid degradation (*ACAA2*, *ACADS*, *ACADM*, *ACSL1*, *CPT1B*, and *CPT2*) as well as the downregulation of genes belonging to the glycolysis/gluconeogenesis and insulin signalling pathways. The systematic increase of *SLC16A1* gene expression during exercise confirmed a significant increase in the intensity of lactate metabolism in horse muscle, which is critical for avoiding lactate accumulation and maintaining tissue homeostasis following repeated bouts of exercise [18]. Additionally, Regatieri et al. [20, 21] indicated a possible association between the MTC1 gene (SLC16A1) and adaptation to physiological stress caused by physical exercise. Moreover, Koho et al. [22] hypothesized that the occurrence of an interaction between the MCT1 and CD147 genes may lead to the activation of lactate transport across the membranes. The disorder of CD147-MCT1 transport complex formation can be associated with the impairment of lactate transport and utilization, leading to the different predispositions to effort observed in individuals. Regatieri et al. [21] also showed differences in allele frequencies for polymorphisms within the Pyruvate Dehydrogenase Kinase 4 (PDK4) gene between Arabian and Quarter horses. Such results may indicate a prospective role of detected SNPs on glucose and fatty acid metabolism regulation as well as performance trait determination.

The research performed in pure-breed Arabian horses during flat racing training periods also showed a significant decrease in the expression of the *SH3RF2* gene, a gene associated with apoptosis. During intensive physical effort, the regulation of cell death by apoptosis can result in the replacement of unaccustomed muscle cells by new cells that are better suited to exercise. Therefore, apoptosis is considered one of the adaptation mechanisms that controls and maintains fitness, whereas muscular strength is considered skeletal tissue remodeling. Analyses of blood transcriptomes confirmed the significant upregulation of interleukins (*IL6ST*, *IL6R*, and *IL7R*) and integrin (*ITGA4*) [19]. This observation confirmed that long-term training is recognized by the organism as a stress factor and that such inflammatory responses can have an important function in triggering downstream signalling pathways, which are critical to homeostasis maintenance [19]. Whole gene expression modification in blood and muscle has allowed the proposal of a panel of genes (*PK3CG*, *FOXO3*, *SLC16A1*, *ME3*, *ACTN3*, *PPARa*, *SH3RF2*, *TPM3*, *TNNC1*, *TNNI3*, *TGFBR1*, *TGFBR2*, and *FABP3*) potentially associated with flat racing performance in Arabian horses; these genes are currently being studied for use as markers for selection [23, 24].

Moreover, based on Arabian horse models, it has been established that monitoring of transcriptomic changes in whole blood can be used as a great tool during identification of osteoclastogenesis [25]. The disturbance of balance between bone resorption and bone formation in response to training overload can be reflected in the modification of gene expression. The research performed on Arabian horses competing at the race track confirmed the significant fluctuation in transcript levels of selected genes strongly related with bone turnover [25]. Among others, the significant modification of *VAV3* (guanine nucleotide exchange factor) and *IL6ST* gene expression (interleukin 6 signal transducer) was observed. Both detected genes are associated with osteoclastogenesis regulation via controlling of osteoclast function (*IL6ST* gene) and osteoclastogenesis enhancement (*VAV3* gene). The quantitative identification of such potential bone turnover markers can influence further research on homeostasis imbalance between bone resorption and formation during fitness achievement in racing Arabian horses leading to early detections of dysfunction in articular cartilage or bone in trained horses. The summary of the founding about genetic background of racing performance in Arabian horses was presented in Table 1.

## 6. Transcriptomic and miRNAomic Analyses of Endurance Capacity in Arabian Horses

To date, the vast majority of genetic analyses exploring racing performance in Arabians has used transcriptomic and miRNAomic methodologies. The modification of gene expression during training was performed in an Arabian horse for the first time in 2013 by Capomaccio et al. [26]. Based on a comparison of the whole transcriptome profile of blood, the authors indicated that exercise was recognized by organisms as a stress factor, and in Arabian horses, intense training significantly affected the transcript levels of integrin genes (*ITGAL*, *ITGAM*) and genes related to protein synthesis (*CXCL1*, *IL1R1*, and *TLR1*), growth factors, or the cell cycle (*EEF1A2*, *GATA2*, *BMP2*, and *FLT4*). The association of interleukins and performance traits was showed previously by Cappelli et al. [27]. The authors, who performed research on Arabian endurance horses using cDNA-AFLP and real-time PCR methods, identified four genes involved in a training-induced stress response: IL8 (interleukin 8), EIF4G3 (eukaryotic translation factor 4 gamma 3), RBBP6 (retinoblastoma binding protein 6), and HSP90AA1 (heat shock protein). Authors showed modification of these gene expression levels under endurance stressing conditions and indicated on their association with exercise stress-induced response.

The comprehensive analysis of mRNA and miRNA expression in the blood of pure-breed or half-breed Arabian horses before and after a 160 km endurance competition has been performed by Mach et al. [28]. The comparative analysis of expression patterns allowed the detection of miRNA and targeted differentially expressed genes related to effort adaptation. The authors identified exercise-induced modifications of genes/miRNAs involved in glucose metabolism, fatty acid oxidation, and immune response pathways that are critical to fitness maintenance. As the most important deregulated genes, authors pinpointed several transcription factors, *ZFP42*, *SPI1*, *FOXO3*, *IRF3*, and *NRF1*, which activated or repressed the expression of miRNAs that play a key role during exercise. Furthermore, research has proposed a panel of miRNAs detected in blood that are strongly associated with endurance effort (miR-21-5p, miR-181b-5p, and miR-505-5p).

A broad range of miRNA effects on gene expression and the translation process have also indicated the necessity for a comprehensive analysis of global gene and miRNA expression profiles in Arabian horses [29, 30]. Mach et al. [28] strongly pinpointed that only investigation of mRNA-miRNA regulation network can give a full information about posttranscriptomic modification during endurance effort. Authors identified miRNAs in the blood of Arabian horses and indicated their significant role in exercise adaptation [28]. Recent studies performed on Warmblood horses showed that exercise affected the expression of 6 miRNAs and 5 targeted genes, which were also identified as differentially expressed under exercise conditions [31]. Kim et al. [31] suggested that such analyses may be useful for elucidating the molecular mechanisms of exercise-associated physiology in horses. Cappelli et al. [32] confirmed that the investigation of the serum profile of miRNAs can lead to the detection of putative biomarkers for exercise adaptation in endurance horses. The authors hypothesized that the identification of miRNAs would have promising results for sports medicine through the discovery of putative biomarkers to predict risks related to prolonged activity and for the monitoring of metabolic adaptations.

The latest studies carried out on Arabian horses before and after a 160 km endurance competition showed how helpful the investigation of blood metabolome, transcriptome, and miRNome can be for providing more complete information on the response to endurance effort [33]. Comparing the differential genes/miRNA expression profiles pre- and postendurance competition and metabolites, authors pinpointed the most important molecular network related to endurance exercise. The comprehensive analysis allowed for detection of 11 metabolites correlated with the expression of 263 metabolic genes and 5 miRNAs. Authors detected five miRNAs (et-7b-5p, miR-16-5p, miR-21-5p, miR-92a-3p, and miR-192-5p) which regulate more than ten metabolic genes and 11 unique metabolites related with the above miRNAs and 263 DEGs. Taking into account the whole metabolome and transcriptome profile, Mach et al. [33] confirmed decreased glucose concentrations after endurance exercise and the metabolic genes belonged to Forkhead box protein O (FoxO) family, which were previously proposed as related to performance traits in Arabians [18, 19]. Mach et al. [33] also indicated deregulation of pentose, propanoate, and glucuronate pathways as well as regulation of the expression of multiple components of the mitochondrial oxidative phosphorylation system (multiple subunits of ATP synthase, ubiquinone oxidoreductase core subunits, and cytochrome oxidase subunits). The metabolomics approach in endurance horses was first applied by Le Moyec et al. [34]. The whole metabolome profiling of plasma allowed for a precise identification of molecules and a more accurate assessment of the amount affected by long endurance exercise. The results clearly indicated a critical role of lipid (correlated with racing speed) and lactate (correlated with ranking position at the end) metabolism. Five years later, Le Moyec et al. were then able to confirm that endurance effort (160 km distance) switches from carbohydrate consumption to lipid consumption. The metabolome modifications are strongly related with distance, training status, and inherited predisposition to effort. Le Moyec et al. [35] also showed that endurance horses at 160 km use the metabolism of fatty acids for energy production and that distance is a primary factor that induces the switch in metabolism (90 to 160 km). The increased request of lipids during training was reflected in the gene expression profiles in blood from Arabian horses during a one-year training schedule [19]. Authors indicated that the improved efficiency of fatty acid utilization was particularly evident between horses in the last training period and untrained horses, where five genes related to lipid metabolism were found as differentially expressed: AGPAT3, AGPAT5, GPD2, LPGAT1, and PCYT1A. The summary of the founding about genetic background of endurance capacity in Arabian horses was presented in Table 2.

## 7. Conclusion

As Arabian horses are mostly endurance performers with directed selection to achieve extraordinary results, additional research conducted in Arabian horses is warranted to identify genetic markers related to endurance traits under selection. The search for a genetic basis of endurance capacity will advance breeding decisions and avoid costly mistakes generated by the mismatched, long-term training of nonpredisposed Arabian horses. Elite endurance performance, which is a characteristic fairly unique to the Arabian breed, is a complex polygenic trait in which comprehensive analyses of genome-wide mutations, gene expression at the transcriptome level, and metabolomics would prove valuable in providing additional information about the genetic basis of endurance exercise phenotypes in not only Arabian horses but all horse breeds.

## Figures and Tables

**Table 1:** Summary of top candidate genes for flat racing performance in Arabian horses.

Gene symbol	Biological function/pathways/gene family	Population	Associated trait(s)	Methodology/type of analysis	Reference
*BCL2*, *FOXO3*, *GNBa*, *ITGA4*, *PIK3AP1*, *PIK3CG*, *ATM*, *FOXO3*, *IL7R*, *STK4*, *AGPAT3*, *AGPAT5*, *GPD2*, *PGAT1*	PI3K-Akt signalling pathway FoxO signalling pathway Notch signalling pathway Lipid metabolism	Pure-breed Arabian horses (Polish population)	General adaptation to effort during flat racing (whole body homeostasis)	Whole transcriptome seq.—RNA-seq (blood tissue)	Ropka-Molik et al., [19]
*SLC16A1*, *FABP3*, *ME3*, *DNER*, *LMCD1*, *LMOD2*, *ANKRD2*, *SH3RF2*, *PPARA*, *ACTN2*, *ACAA2*, *LPL*	Regulation of actin cytoskeleton, fatty acid degradation, PPAR signalling pathway, lactate metabolism	Pure-breed Arabian horses (Polish population)	General adaptation to effort during flat racing (muscle tissue remodeling, body homeostasis)	Whole transcriptome seq.—RNA-seq (muscle tissue)	Ropka-Molik et al., [18]
*MCT1 (SLC16A1)*, *CD147*, *PDK4*, *DMRT3*	Lactate transport and metabolism, glucose and fatty acid metabolism, limb movement regulation	Pure-breed Arabian	Possible association of PDK4 and energy production, DMRT3, and gait type	Sanger sequencing ARMS-PCR, PCR-RFLP	Regatieri et al., [21]
*SH3RF2*	Antiapoptotic regulator of the JNK pathway	Pure-breed Arabian horses (Polish population)	Flat racing performance (winning, number of starts)	Whole transcriptome seq.—RNA-seq, PCR-RFLP (muscle tissue)	Ropka-Molik et al., [23]
*BGLAP*, *CTSK*, *TYROBP*, *PDLIM7*, *SLC9B2*, *TWSG1*, *NOTCH2*, *IL6ST*, *VAV3*, *NFATc1*, *CLEC5A*, *TXLNG*, *TCAP*	Regulation of actin cytoskeleton, osteoclast differentiation, glycerophospholipid metabolism	Pure-breed Arabian horses (Polish population)	Potential bone turnover markers, bone homeostasis indicators	Whole transcriptome seq.—RNA-seq, qPCR	Stefaniuk-Szmukier et al., [25]
*ACTN3*	The alpha-actin binding protein gene family structural component of sarcomeric Z line	Pure-breed Arabian horses (Polish population)	Potentially associated with flat racing performance	Whole transcriptome seq.—RNA-seq, qPCR (muscle tissue)	Ropka-Molik et al., [24]

**Table 2:** Summary of top candidate genes for endurance ability in Arabian horses.

Gene/miRNA symbol	Biological function/pathways/gene family	Population	Associated trait(s)	Methodology/type of analysis	Reference
*IL8*, EIF4G3, HSP90AA1, RBBP6	Interleukins, heat shock proteins	Arab endurance horses	Training-induced stress response	cDNA-AFLP RT-PCR	Cappelli et al., [27]
*ITGAL*, *ITGAM*, IL22A2, *CXCL1*, *IL1R1*, *TLR1*, *EEF1A2*, *GATA2*, *BMP2*, *FLT4*, MAP3K4, MAPK14	Growth factors or the cell cycle regulation molecules, integrin, interleukins, kinases	Pure-breed Arabian horses	Inflammation and immune system activation occur following physical stress in equine athletes	SOLiD whole transcriptome (blood tissue)	Capomaccio et al. [26]
*ZFP42*, *SPI1*, *FOXO3*, *IRF3*, *NRF1*, *miR-21-5p*, *miR-181b-5p*, *miR-505-5p*	FoxO signalling pathway, peroxisome proliferator-activated receptor (PPAR*γ*) signalling pathway, oxidation stress, proteolysis and immune response pathways	Pure-breed or half-breed Arabian	Glucose metabolism, fatty acid oxidation, and immune response exercise adaptation	RNA and miRNA microarrays (blood tissue)	Mach et al., [28]
*SORCS3*, *SLC39A12*, *KCNQ1OT1*	Mitochondrial metabolism, oxidative phosphorylation metabolism, metal ion, cation binding, and haematopoiesis	Pure-breed Arabians and Arabian-cross horses (French population)	Total race distance, average race speed, finishing status, cardiac rhythm	GWAS	Ricard et al., [36]
*TLR4*, *FoxO3*, CREBBP, RBL2, SIRT1, BCL6, POLR2I, TOR3A, *miR-92a-3p*, *miR-192-5p*, *miR-21-5p*, *miR-16-5p*, *and let-7 family*	LPS-induced proinflammatory pathway; energy-purine and pyrimidine catabolism	Pure-breed or half-breed Arabian horses	Physiological adaptation to physical exertion; regulation of metabolic and immune response to endurance exercise	Transcriptome and miRNome NGS sequencing, metabolome (blood tissue)	Mach et al., [33]
*miR-1*, *miR-133a-3p*, *miR-133b*, *miR-133a*, *miR-206*, *miR-208b*, *miR-224*, *miR-95*, *miR-1180*, *miR-499b-3p*, *miR-486-3p*, *miR-486-5p*, *miR-504-5p*	Targeted genes involved in muscle remodeling (EGFR, IGF1, PURB, TAGLN), cellular homeostasis maintenance (SLC5A3, SLC1A2, SLC7A1)	Pure-breed Arabian horses	Biomarkers for exercise adaptation to endurance effort	miRNAome NGS sequencing (blood tissue)	Cappelli et al., [32]
